# Sorting Sox: Diverse Roles for Sox Transcription Factors During Neural Crest and Craniofacial Development

**DOI:** 10.3389/fphys.2020.606889

**Published:** 2020-12-08

**Authors:** Elizabeth N. Schock, Carole LaBonne

**Affiliations:** ^1^Department of Molecular Biosciences, Northwestern University, Evanston, IL, United States; ^2^NSF-Simons Center for Quantitative Biology, Northwestern University, Evanston, IL, United States

**Keywords:** neural crest, craniofacial development, SoxB1, SoxE, stem cells

## Abstract

Sox transcription factors play many diverse roles during development, including regulating stem cell states, directing differentiation, and influencing the local chromatin landscape. Of the twenty vertebrate Sox factors, several play critical roles in the development the neural crest, a key vertebrate innovation, and the subsequent formation of neural crest-derived structures, including the craniofacial complex. Herein, we review the specific roles for individual Sox factors during neural crest cell formation and discuss how some factors may have been essential for the evolution of the neural crest. Additionally, we describe how Sox factors direct neural crest cell differentiation into diverse lineages such as melanocytes, glia, and cartilage and detail their involvement in the development of specific craniofacial structures. Finally, we highlight several SOXopathies associated with craniofacial phenotypes.

## Introduction

Since the hallmark discovery of the *SRY* gene, the master regulator of male sex determination (Gubbay et al., 1990; Lovell-Badge, 2010), twenty mammalian SRY-related HMG box containing (SOX) transcription factors have been identified. A significant number of important developmental functions have been described for Sox transcription factors. These range from maintaining stem cell states to promoting differentiation (reviewed in Kamachi and Kondoh, 2013). The growing number of developmental disorders associated with mutations in *SOX* genes underscores the importance of these factors during development (Angelozzi and Lefebvre, 2019). In this review we focus on the Sox factors that have roles in the formation of the neural crest as well as those important for the development of cell types within and components of the craniofacial complex. Finally, we highlight the “SOXopathies” that are associated with a variety of craniofacial phenotypes.

## Sox Transcription Factor Families

**FIGURE 1:**
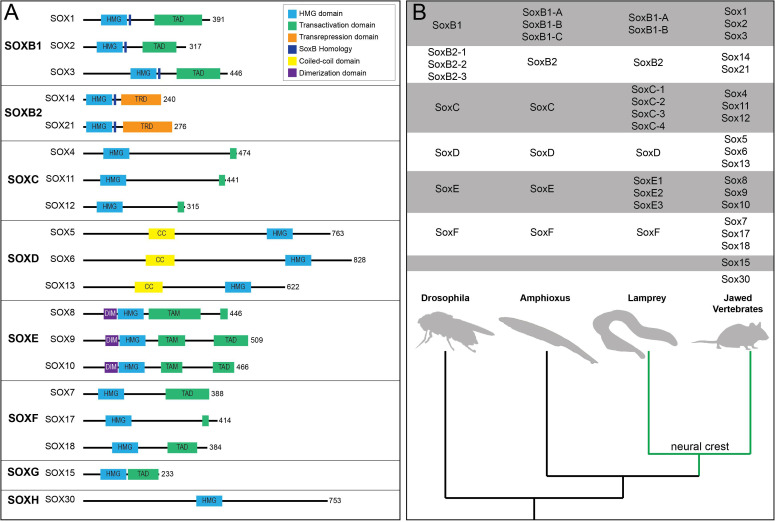
Sox transcription factor subfamiles and their functional domains. **(A)** Diagram of human SOX proteins grouped by subfamily. Major protein functional domains are depicted as colored boxes: High-mobility group (HMG) domain (light blue), transactivation domain (TAD; green), transrepression domain (TRD; orange), SoxB homology domain (dark blue), coiled-coil (CC) domain (yellow), and dimerization (DIM) domain (purple). **(B)** Phylogenetic tree depicting Sox subfamily members across key organisms. The emergence of the neural crest (green) in vertebrates coincided with duplication events among various Sox subfamilies, most notably SoxC and SoxE.

The twenty mammalian Sox factors are divided into nine subfamilies (A, B1, B2-H) based mainly upon the homology of a 79 amino acid DNA binding region termed the High Mobility Group (HMG) domain (Figure 1A; Bowles et al., 2000). Duplication events, slow divergence, and co-option of functional elements are hypothesized to have driven Sox family evolution (Bowles et al., 2000). Consistent with this, members of the same subfamily often having overlapping expression patterns and various degrees of functional redundancy (Figure 1B; Heenan et al., 2016). SOX factors bind DNA at C[A/T]TTG[T/A][T/A] sequences or similar motifs. Notably, the HMG domain binds DNA at the minor groove causing the DNA to bend. This facilitates local chromatin modifications and can increase DNA accessibility for partner factor binding (Hou et al., 2017). Furthermore, some SOX factors (SOX2 and SOX9) have been shown to engage at regions of condensed chromatin and are considered pioneer factors (Adam et al., 2015; Soufi et al., 2015; Julian et al., 2017). SOX2, through the HMG domain, recognizes a degenerate Sox motif on nucleosomal DNA (Soufi et al., 2015). At these degenerate sites less DNA bending occurs which facilitates SOX2 binding on the minor groove of nucleosomal DNA (Soufi et al., 2015). At some of these sites, binding may, in part, be facilitated or stabilized by chromatin-associated proteins such as poly(ADP-ribose) polymerase-1 (PARP-1) (Liu and Kraus, 2017). The extent to which SOX9’s pioneer activity mechanistically mimics that of SOX2 is not known and is an area ripe for future investigation. In order to activate gene expression, SOX factors generally require cooperation with a partner factor (reviewed in Kondoh and Kamachi, 2010). Examples of this include SOX10-MITF pairing during melanocyte specification, SOX2-OCT3/4 pairing in embryonic stem cells, and SOX2-BRN2 pairing in neural progenitors (Ambrosetti et al., 1997; Jiao et al., 2004; Tanaka et al., 2004; Takahashi and Yamanaka, 2006). Sox factors, other than the SoxB2 subfamily, were initially characterized as transcriptional activators, however, it has since been shown that many Sox factors can function as either activators or repressors in a context-dependent manner (reviewed in Chew and Gallo, 2009).

Within a Sox subfamily, the structural domains of the proteins outside of the HMG domain are similar, but not identical. These domains include coiled-coiled, dimerization, and transactivation/transrepression domains (Figure 1A). SoxD factors harbor a coiled-coil domain that mediates homo- or hetero-dimerization with other SoxD factors, stabilizing binding to adjacent HMG sites on DNA (Lefebvre et al., 1998). SoxE factors possess a 40 amino acid dimerization (DIM) domain upstream of the HMG domain (Peirano and Wegner, 2000). Their dimerization (homo- or hetero-) is DNA dependent and reliant upon the presence of a palindromic DNA binding sequence (Peirano and Wegner, 2000; Peirano et al., 2000; Schlierf et al., 2002; Huang et al., 2015). While SOXE factors can form heterodimers, they do not appear to dimerize with non-SoxE proteins (Huang et al., 2015; Hou et al., 2017). SoxE factors additionally possess both a C-terminal transactivation domain (TAD) and a second transactivation domain in the middle of the protein (TAM, or K2 domain) (Schreiner et al., 2007). Recent work suggests that these two domains synergize, resulting in a SOXE factor bipartite transactivation mechanism (Haseeb and Lefebvre, 2019). The members of other Sox subfamilies possess a single transactivation/repression domain. Interestingly, Sox transcriptional activity can depend upon whether the transcriptional partner is an activator or repressor (Kamachi and Kondoh, 2013). For example, SOX2-OCT3/4 synergistically activate *Fgfr4* expression and the SOX9-SOX5/6 complex activates *Col2a1* during chondrogenesis (Ambrosetti et al., 1997; Liu and Lefebvre, 2015). In contrast, SOX9-GLI2/3 represses *Col10a1* in non-hypertrophic chondrocytes (Leung et al., 2011). SOX proteins also associate with non-DNA binding cofactors, such as Groucho co-repressors. SOX2-GRG5 represses neural differentiation markers and SOX9-GRG4 represses *Dct* expression during melanocyte development (Lee et al., 2012; Liu et al., 2014). These findings highlight the importance of cellular context and partner protein/cofactor availability for Sox function.

Sox factors have diverse roles during development and typically members of the same subfamily have similar or redundant functions. The contributions of SoxB1 factors to maintaining pluripotency have been intensely studied (reviewed in Abdelalim et al., 2014), and SOX2 is one of the four Yamanaka factors able to reprogram somatic cells to a pluripotent state (Takahashi and Yamanaka, 2006). Interestingly, other subfamilies of Sox factors are capable of replacing SoxB1 function either during the reprogramming process or in stem cells. SOX15 and SOX18 can substitute for SOX2 during the reprogramming process, but are less efficient (Nakagawa et al., 2008). While SOX17 is not an effective substitute for SOX2, a reengineered SOX17 (SOX17 E122K) reprograms cells with high efficiency (Jauch et al., 2011). Likewise, the reprogramming efficiency of SOX18 increases when it is reengineered to have an analogous point mutation within the HMG domain and the C-terminal of the protein is swapped for the SOX17 C-terminal (Aksoy et al., 2013). The point mutation within the SOX18 HMG domain alone was not sufficient for reprogramming nor was swapping the C-terminal for that of SOX2 (Aksoy et al., 2013). With respect to regulating a stem cell state in embryos, morpholino mediated knockdown of Sox2 and Sox3 in *Xenopus* leads to a loss of pluripotency and this can be partially rescued by expression of *SoxE* factors (Buitrago-Delgado et al., 2018). Together these data suggest that while specific subfamilies of Sox factors may be optimized for particular developmental roles, other Sox subfamilies may be able to serve as a substitute, albeit less efficiently. This paradigm is particularly interesting in the context of the neural crest and the retention of embryonic potential in those cells (discussed below; Buitrago-Delgado et al., 2015).

## Roles for Sox Factors During Neural Crest Formation

**FIGURE 2:**
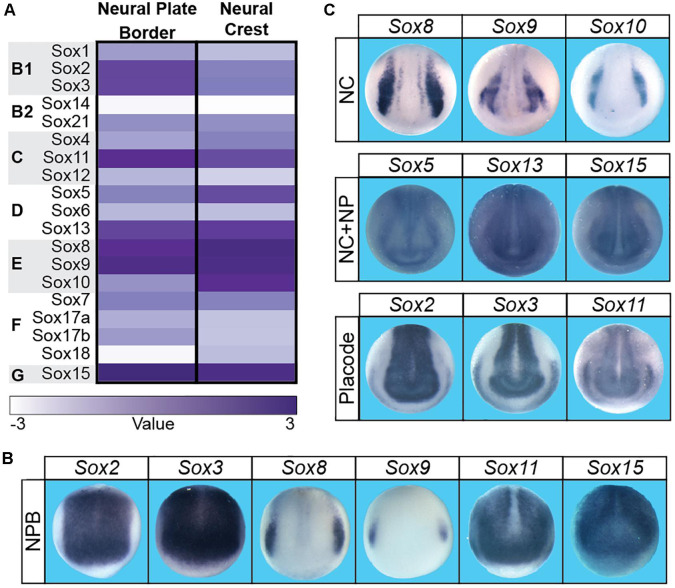
There were boxes subdividing the three sections **(A–C)** in the submitted figure. These boxes are not present in this proof. *Sox* factor expression during neural plate border and neural crest formation in *Xenopus laevis.* **(A)** Heatmap of *Sox* gene expression in neural plate border and neural crest cells in *Xenopus laevis*. High levels of expression are associated with a dark purple while low levels are depicted as white. **(B)** *In situ* hybridizations in early neurula *Xenopus laevis* embryos for *sox2*, s*ox3*, s*ox8*, s*ox9*, s*ox11*, and s*ox15*. Each of these factors has neural plate border expression (domain most clearly defined by s*ox8* expression). **(C)** *In situ* hybridizations in late neurula *Xenopus laevis* embryos for *sox f*actors expressed in the neural crest (*sox8*, s*ox9*, s*ox10*), neural crest + neural plate (*sox5*, s*ox13, sox15)*, and neural plate + placodes (*sox2, sox3, sox11*).

The neural crest is a vertebrate specific population of cells that contribute significantly to the vertebrate body plan, including much of the craniofacial complex. In addition to giving rise to the bone and cartilage of the face, the neural crest also give rise to melanocytes, the majority of the peripheral nervous system, and contribute directly to facial structures such as the tongue, teeth, and palate (Chai et al., 2000; Haldin and LaBonne, 2010; Bronner and LeDouarin, 2012; Prasad et al., 2012). Embryonically, neural crest cells (and cranial placodes) arise at the neural plate border which lies between the neural plate and non-neural ectoderm. Gradients of BMP, FGF, and WNT signaling have all been implicated in the establishment of these three regions. The initial formation of the neural plate border occurs in cells with intermediate levels of BMP signaling and high WNT signaling. A unique signature of transcription factors (*pax3/7*, *zic1/2*, *msx1*, *myc*) define the neural plate border region and these factors subsequently activate the neural crest gene regulatory network (GRN), which includes several Sox factors (Simoes-Costa and Bronner, 2015). *Sox2*, *sox3*, *sox8*, *sox9*, *sox11*, and *sox15* are expressed within the neural plate border (Figure 2; Spokony et al., 2002; Wakamatsu et al., 2004; O’Donnell et al., 2006; Roellig et al., 2017). *SOX2* expressing cells within the neural plate border can contribute to both the neural crest and the non-neural ectoderm, and modulating levels of *SOX2* can impact the balance between neural and neural crest domains (Roellig et al., 2017). The observed expression of *sox8* and *sox9* correlates with the formation of definitive neural crest cells within the neural plate border.

### Sox Subfamily Function During Neural Crest Formation

Sox transcription factors play important roles in controlling the developmental potential of the neural crest progenitor population as well as in their subsequent lineage decisions. Members of the *SoxC*, *SoxG*, *SoxD*, and *SoxE* families are expressed in the pre-migratory neural crest and functional roles for most of these factors have been reported (Figure 2). By contrast, *SoxB1* factors are expressed in the neural plate and pre-placodal ectoderm and can inhibit neural crest formation (Wakamatsu et al., 2004; Roellig et al., 2017; Buitrago-Delgado et al., 2018). *SoxC* factors (*sox4/11/12*) are expressed in the neural crest and the neural plate of *Xenopus* and lamprey (*Petromyzon marinus*) embryos and seem to have evolutionarily conserved functions. Loss of function experiments for SoxC factors resulted in failed neural crest formation in both species (Uy et al., 2015). *SoxC* factors are also expressed in migrating neural crest, but a requirement for these factors during migration or subsequent lineage diversification has yet to be described (Cizelsky et al., 2013; Uy et al., 2015). Similarly, the SoxG family member, *Sox15*, is expressed in the pre-migratory neural crest, but a role for Sox15 in neural crest development has yet to be reported. Interestingly, *Sox15* is expressed in mouse embryonic stem cells and, like SOXB1 proteins, can associate with OCT3/4 (Maruyama et al., 2005). While *Sox15*-null mice are viable, it is possible that SOX15 function is redundant to that of SOXB1 factors in ES cells. As neural crest cells retain stem-cell like potential, it would be interesting to investigate if SOX15 plays a role in controlling the developmental potential of these cells (Buitrago-Delgado et al., 2015).

Of the three SoxD family members, *Sox5* and *Sox13* are expressed in the forming neural crest; however, only a role for SOX5 has been reported in these cells. *SOX5* is expressed in the pre-migratory neural crest in both chick and *Xenopus* embryos. Loss of function experiments demonstrated that Sox5 is necessary for neural crest, placode, and neural plate border formation. Interestingly, increasing Sox5 levels phenocopies these effects (Nordin and LaBonne, 2014), suggesting that maintaining the correct level of *sox5* expression is key to proper neural crest formation. Sox5 can serve as an effector of BMP signaling in the ectoderm (and in other biological contexts). Through its central coiled-coil domain, Sox5 physically interacts with BMP R-Smad complexes and promotes activation of BMP target genes (Nordin and LaBonne, 2014). Since BMP signaling is essential for neural plate border/neural crest formation, Sox5 likely aids in activation of BMP targets in neural plate border/neural crest cells. Expression of *sox5* persists as neural crest cells migrate, and overexpression of *sox5* results in both a delay in migration and an increase in the total number of neural crest cells (Perez-Alcala et al., 2004; Nordin and LaBonne, 2014). Whether this increase is at the expense of other cell types in the ectoderm remains to be determined.

The evolutionary emergence of the neural crest correlated with the duplication, diversification, and neofunctionalization of a single ancestral *SoxE* gene (Tai et al., 2016). Where it has been examined, vertebrates possess two/three *SoxE* paralogs (Tai et al., 2016). SoxE factors have been shown to be required for neural crest formation across multiple species (Spokony et al., 2002; Honore et al., 2003; Hong and Saint-Jeannet, 2005; Taylor and LaBonne, 2005; O’Donnell et al., 2006; Buitrago-Delgado et al., 2018). Interestingly, the temporal order of *SoxE* gene expression within the neural crest differs across species. In *Xenopus*, *sox8* is expressed first followed by *sox9* then *sox10* (O’Donnell et al., 2006; Buitrago-Delgado et al., 2018). In chick and mouse, *Sox9* is the first SoxE factor to be expressed within the neural crest followed by *Sox10* then *Sox8* (Cheung and Briscoe, 2003). In zebrafish, *sox9a* and *sox9b* expression precedes that of *sox10* in the neural crest while *sox8* is not expressed in these cells (Dutton et al., 2001; Li et al., 2002; Yan et al., 2005). The varied timing of *Sox* expression is consistent with a high degree of functional redundancy among these factors. In the agnathan, *Petromyzon marinus, SoxE1* and *SoxE2* are expressed in the neural folds and migrating neural crest while *SoxE3*, the ortholog to *Sox9* in gnathostomes, lacks early embryonic expression (McCauley and Bronner-Fraser, 2006). The differences in order of expression and function between agnathan and gnathostome SoxE factors may suggest independent divergence from the ancestral *SoxE* (Lakiza et al., 2011).

Evidence across multiple organisms suggests that while SoxE factors are required for neural crest formation, the individual factors may be functionally redundant. Loss of function studies in *Xenopus* demonstrated that *sox8, sox9*, and *sox10* are necessary for neural crest formation and proper migration (Spokony et al., 2002; Honore et al., 2003; Taylor and LaBonne, 2005; O’Donnell et al., 2006). The neural crest can be rescued in Sox8 morphants by any of the SoxE factors suggesting there is functional redundancy between the SoxE factors (Taylor and LaBonne, 2005; O’Donnell et al., 2006). By contrast, murine SoxE mutants do not have obvious early neural crest defects. *Sox8* null mice are viable with no obvious morphological defects suggesting that during mammalian development the other SoxE factors are able to compensate for loss of *Sox8* (Sock et al., 2001). Additionally, the neural crest in *Sox9^*fl/fl;*^Wnt1Cre* embryos migrates normally to the craniofacial complex; this suggests that neural crest formation is not altered in these conditional mutants; however, increased apoptosis has been observed in the trunk neural crest of *Sox9*^–/–^ mice (Cheung et al., 2005). The *Sox9^*fl/fl;*^Wnt1Cre* mice do, however, have major craniofacial defects, as discussed below (Mori-Akiyama et al., 2003). In *Sox10^*lacZ*^/Sox10^*lacZ*^* mice and *Sox10* hypomorphs, neural crest formation and migration is not altered (Britsch et al., 2001; Schreiner et al., 2007). Again, this suggests that the other SoxE factors can compensate for the loss of a single SoxE factor during neural crest formation. In contrast, during neural crest cell differentiation, each SoxE factor may have unique functions. This is evidenced by *Sox10^*Sox*8*ki/Sox*8*ki*^* embryos, where *Sox8* has been inserted into the *Sox10* locus. These mice still have severe enteric nervous system defects and lack melanocytes which phenotypically parallel a loss of *Sox10* (Kellerer et al., 2006).

### Post-translational Modifications of SOX Proteins in the Neural Crest

SOX proteins undergo a number of post-translational modifications, including: acetylation, methylation, phosphorylation, SUMOylation, and ubiquitination (Williams et al., 2019). Several of these modifications have been shown to impact neural crest development (Huang et al., 2000; Taylor and LaBonne, 2005, 2007; Sakai et al., 2006; Lee et al., 2012; Liu et al., 2013). Phosphorylation of SOX9 at serine 64 and serine 211, conserved residues in amniotes, by PKA increased SOX9 DNA-binding affinity at a *Col2a1* enhancer element and promoted reporter activation (Huang et al., 2000). Furthermore, phosphorylation of the analogous serine residues in chick is required for delamination of trunk neural crest cells (Liu et al., 2013). Delamination is mediated through SOX9-SNAI2 interaction, and phosphorylation of SOX9 is required for this pairing. Additionally, SOX9 serine 64 and serine 181 phosphorylation (in chick) promotes SOX9 SUMOylation (Liu et al., 2013). SOX9 SUMOylation is not required for trunk delamination, but SUMOylation state impacts neural crest cell formation (Taylor and LaBonne, 2005; Liu et al., 2013). Blocking Sox9 SUMOylation promotes neural crest formation whereas constitutively SUMOylated Sox9 represses neural crest formation (Taylor and LaBonne, 2005). Sox9 inhibition of the neural crest state is mediated through SUMO-dependent recruitment of the Groucho family protein Grg4, which is a transcriptional co-repressor (Lee et al., 2012). The SUMOylation state of Sox9 also influences inner ear development. A constitutively SUMOylated form of Sox9 promotes otic vesicle formation whereas a form of Sox9 that cannot be SUMOylated inhibits ear formation, but promotes ectopic melanocytes (Taylor and LaBonne, 2005).

Likewise, SOX10 is SUMOylated at three conserved lysine residues (Taylor and LaBonne, 2005; Girard and Goossens, 2006). SUMOylation state of SOX10 does not impact nuclear localization or the ability of SOX10 to bind DNA; however, it inhibits the transcriptional activation of target genes such as *MITF* (Girard and Goossens, 2006). Furthermore, it was demonstrated that SUMOylation interferes with the ability of SOX10 to synergize with PAX3 to activate *MITF* gene expression. This was also true for SOX10 and ERG2 and their target *GJB1* in Schwann cells (Girard and Goossens, 2006). Whether this relationship between SOX10 SUMOylation state and transcriptional activation is true for all target genes is yet to be determined. It is likely, however, that this relationship is more complex and context dependent as observed with other transcription factors (Long et al., 2004; Taylor and LaBonne, 2005; Rosonina et al., 2017). Finally, SoxE factors also have putative acetylation and methylation sites (Williams et al., 2019); however, functional roles for these modifications during neural crest development remains largely unknown. One study demonstrated that Sox9 is acetylated by Tip60; however, the acetylation state did not impact the ability of Sox9 to activate *Col2a1* expression (Hattori et al., 2008). Whether Sox9 acetylation state affects transcription of other target genes or if other Sox9 post-translational modifications regulate *Col2a1* expression are questions that remain to be answered.

### SOX Transcriptional Targets in the Neural Crest

The functional relationship between SoxE factors and other transcription factors essential for neural crest formation has been examined in several systems. Studies in *Xenopus h*ave shown that Snail1 promotes *sox10* expression and knockdown of *sox9* leads to reduced *twist*, *snail1* and *pax3* expression in the neural crest, whereas *sox9* or *sox10* gain-of-function expands the domains of *foxD*3, *snail2*, and *sox10* expression at the neural plate border (Spokony et al., 2002; Honore et al., 2003; Buitrago-Delgado et al., 2018). In chick, overexpression of *SOX9* is not sufficient to induce *PAX3/7*, but can induce *SNAI2, FOXD3*, and *SOX10* (Cheung and Briscoe, 2003). Additionally, a *SOX10* enhancer element has been identified in chick which requires ETS1, SOX9, and/or cMYB activity to drive reporter expression (Betancur et al., 2010). While such candidate-driven functional studies have provided some insights into the functions of SoxE factors within the neural crest gene regulatory network, a more comprehensive understanding of Sox targets in the neural crest remains lacking. Moreover, as SOX factors require DNA-binding partners for efficient regulation of target genes, it is also essential to identify and study the SOXE partners that play roles in the development and evolution of the neural crest.

### SoxE Factors and the Retention of Embryonic Potential in the Neural Crest

Evolutionarily, the emergence of the neural crest coincided with duplication and diversification of an ancestral *SoxE* gene (Figure 1B; Tai et al., 2016). The significance of SoxE function to neural crest evolution is interesting to contemplate given the central role these factors play in the establishment of the neural crest stem cell state. As SoxB1 factors are essential regulators of pluripotency in blastula cells, it is possible that this role was handed off to SoxE factors in the neural crest. Consistent with this, recent work has shown that SoxE function can at least partially replace SoxB1 factors in maintaining the pluripotency of blastula stem cells, although SoxB1 factors are unable to replace SoxE factor function in the neural crest (Buitrago-Delgado et al., 2018). These data suggest that SoxE factors can engage in the pluripotency gene regulatory network, maintaining expression of key targets in the absence of SoxB1 factors, even in a cellular context in which they are normally not expressed. Whether this is a unique feature of SoxE factors or if other Sox subfamilies can function in a similar context remains to be seen; however, other Sox factors can substitute for SoxB1 factors during cell reprogramming (Nakagawa et al., 2008; Jauch et al., 2011). Interestingly, SOX17 sits at the top of the specification hierarchy for human primordial germ cells (Irie et al., 2015). Other pluripotency genes, such as *NANOG* and *OCT4*, but not *SOX2*, are also expressed in human primordial germ cells downstream of *SOX17* (Tan and Tee, 2019). Thus, the SoxB1 to SoxE hand-off in the neural crest may serve as a paradigm for transitioning molecular regulatory circuitry from one Sox subfamily to another to maintain a stem cell-like state.

Why might a transition from SoxB1 to SoxE function have been important for the evolutionary emergence of neural crest stem cells? By the end of gastrulation SoxB1 factors cease to direct pluripotency and instead are expressed in, and essential to, the formation of the neuronal progenitor pool, and continue to play prominent roles in neural lineages later in development (Rex et al., 1997; Graham et al., 2003; Ellis et al., 2004; Schlosser et al., 2008). Once SoxB1 factors have transitioned to controlling the neuronal progenitor state, SoxE factors may take over the regulation of targets essential to maintaining developmental potential in the neural crest. Furthermore, the inability of SoxB1 factors to replace SoxE factor function in the neural crest implies this switch was necessary for the emergence of the neural crest and its derivatives (Buitrago-Delgado et al., 2018). Mechanistically, it is possible that SoxB1 factors are unable to correctly regulate some SoxE target genes unique to neural crest. Furthermore, this switch in Sox factor deployment may also have facilitated the subsequent lineage diversification of neural crest cells to non-neural cell types including cartilage, melanocytes, and glia, which require SoxE function for their formation (Bi et al., 1999; Britsch et al., 2001; Aoki et al., 2003). Understanding this transition, as well as why SoxE factors play essential roles in directing the development of only a subset of neural crest lineages, will require a more complete understanding of SoxE targets and partners in both neural crest stem cells and their derivatives.

## Sox Factors and Development of the Craniofacial Complex

An excellent context in which to investigate the roles and regulation of Sox factors in neural crest lineage diversification is the craniofacial complex, a compilation of multiple structures that together create both the form and function of the face. While development of many of these structures occur simultaneously, the molecular mechanisms that govern their development are unique. The neural crest contributes to a significant portion of the craniofacial complex, giving rise to chondrocytes, melanocytes, a majority of the peripheral nervous system, and contributing to the mesenchymal component of structures such as the tooth and palate (Chai et al., 2000; Bronner and LeDouarin, 2012). Several Sox factors are expressed throughout the facial ectoderm and mesenchyme (Table 1), many of which play essential roles in the development of specific craniofacial structures. Other Sox factors, while expressed during craniofacial development, have yet to be functionally characterized. SoxE functions within neural crest-derived cell types of the face are of particular evolutionary significance. The basal chordate amphioxus possesses a single SoxE gene and lacks neural crest cells, yet has an oral skeleton (Jandzik et al., 2015). Interestingly, expression of amphioxus SoxE within the chick neural tube is sufficient to induce neural crest formation. These amphioxus SoxE expressing cells later expressed markers for DRG lineages, including those cells positioned dorsolaterally that would typically become melanocytes (Tai et al., 2016). These data suggest that co-option of SoxE to proto neural crest-like cells may have occurred prior to genome duplication, but required the aquisition of new *cis-*regulatory sequences (Jandzik et al., 2015; Tai et al., 2016). It further suggests that duplication and divergence of the SoxE family was necessary for neural crest lineage diversification and the subsequent elaboration of the vertebrate head.

**TABLE 1:** *Sox* gene expression in various craniofacial structures in mouse embryos.

Sox Family	Gene	Cartilage	Palate	Teeth	Tongue
B1	Sox1	n.e.	n.e.	n.e.	Muscle
	Sox2	n.e.	Epithelium	Epithelium	Epi + Mus
	Sox3	n.e.	n.e.	n.e.	n.e.
B2	Sox14	n.e.	n.e.	Epithelium	n.e.
	Sox21	n.e.	n.e.	Epithelium	Epi + Mes + Mus
C	Sox4	Yes (broadly Mes)	Mesenchyme	Epi + Mes	Epi + Mes + Mus
	Sox11	n.e.	Epi + Mes	Epi + Mes	Epi + Mes + Mus
	Sox12	n.e.	Epi + Mes	Epi + Mes	Epi + Mes + Mus
D	Sox5	Yes	Mesenchyme	Mesenchyme	Epi + Mes
	Sox6	Yes	Epi + Mes	Epi + Mes	Epi + Mes + Mus
	Sox13	n.e.	Epi + Mes	Epi + Mes	Mes
E	Sox8	n.e.	Mesenchyme	Epi + Mes	Mes + Mus
	Sox9	Yes	Mesenchyme	Epi + Mes	Epi + Mes + Mus
	Sox10	n.e.	n.e.	n.e.	Mes + Mus
F	Sox7	n.e.	n.e.	n.e.	n.e.
	Sox17	n.e.	n.e.	Epithelium	n.e.
	Sox18	n.e.	n.e.	n.e.	n.e.
G	Sox15	n/a	n/a	n/a	n/a

*More detailed expression patterns for factors can be found in: (Kawasaki et al., 2015; Watanabe et al., 2016; Ishikawa et al., 2018). Epithelial (Epi), Mesenchymal (Mes), Muscle (Mus), Not expressed (n.e).*

### Neural Crest Derivatives

#### Sox Factors and Craniofacial Bone and Cartilage

The craniofacial skeleton serves as the framework for the face. The bones of the craniofacial complex differ from long bones, such as those of the arms, legs, or ribs, in two main ways. First, a majority of anterior craniofacial bones are derived from the neural crest while long bones are derived from the mesoderm (Noden and Trainor, 2005). Second, long bones form through a process called endochondral ossification, whereas the flat bones of the face and the bones of the skull form through intramembranous ossification (Berendsen and Olsen, 2015). The small subset of endochondral facial bones included the malleus, incus, and nasal capsule (Mori-Akiyama et al., 2003). These and the facial cartilages (such as Meckel’s) form through a chondrogenic mechanism that requires Sox transcription factors.

SOX9 serves as the central transcriptional regulator for chondrogenesis (Bell et al., 1997; Bi et al., 1999). In vertebrates, *Sox9* is expressed in the neural crest-derived facial mesenchyme (Wright et al., 1995; Healy et al., 1996; Spokony et al., 2002). Heterozygous *Sox9* knock-out mice display cleft secondary palate, domed skull, and a short snout (Akiyama et al., 2002). Conditional deletion of *Sox9* in the neural crest (*Sox9^*fl/fl*^;Wnt1-Cre*) results in complete loss of facial cartilages and endochondral derived bones (Mori-Akiyama et al., 2003). Likewise, in *Xenopus*, Sox9 morphants display gross morphological defects in their craniofacial chondrogenic elements, including complete loss of Meckel’s cartilage (Spokony et al., 2002). In the absence of *Sox9*, the prechondrogenic mesenchymal condensation fails to form and *Col2a1*, a direct target of SOX9, fails to be expressed (Lefebvre et al., 1997; Mori-Akiyama et al., 2003). The cells that should have become chondrocytes begin to express osteoblast markers (such as *Runx2*), indicating that SOX9 not only functions to promote chondrogenesis, but also inhibits osteoblast formation (Mori-Akiyama et al., 2003). Additionally, SOX9 is required for the expression of *Sox5* and *Sox6* during chondrogenesis (Akiyama et al., 2002). *Sox5*^–/–^ and *Sox6*^–/–^ mutants experience early postnatal lethality, but do not have major defects in chondrogenic elements. Interestingly, while *Sox5*^–/–^ mice display minor defects in the craniofacial cartilage; *Sox6*^–/–^ mutants do not. In contrast, *Sox5*^–/–^;*Sox6*^–/–^ double mutants die at e16.5 and fail to form cartilages. While these mutants still express *Sox9* and *Col2a1* in prechondrogenic regions, cells fail to differentiate into chondrocytes (Smits et al., 2001). These data indicate that SoxD factors have both unique roles and redundant roles during chondrogenesis. Analysis of DNA occupancy by SOX9 and SOX5/6 in chondrogenic cells indicates shared binding at enhancers and suggests that SOX5/6 act cooperatively with SOX9 to promote gene activation (Liu and Lefebvre, 2015). Indeed, the triple combination of SOX9-SOX5-SOX6 can promote chondrogenesis in mesenchymal stem cell without the addition of growth factors, such as TGFβ3 (Raftery et al., 2020).

#### Sox Factors and Melanocytes

**FIGURE 3:**
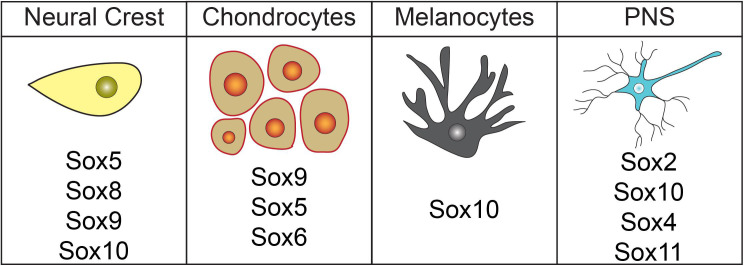
Sox factors involved in neural crest formation and lineage diversification. Diagram highlighting Sox factors known to have roles in neural crest cell, chondrocyte, melanocyte, and peripheral nervous system development.

The pigment of the skin, hair, and choroid layer of the eye is produced by melanocytes. These cells are derived from the neural crest and localize to the vascular uvea of the eye, the basal layer of the epidermis, or hair follicles (Hirobe, 1984; Holbrook et al., 1989; Sitiwin et al., 2019). Sox10 plays a central role in the gene regulatory network (GRN) controlling melanocyte development (Figure 3), although Sox5, Sox9, and Sox18 may also play minor roles in melanogenesis (reviewed in Harris et al., 2010). *Sox10*, first expressed in the neural crest cells prior to migration, persists in the neural crest cells that become melanocytes (Aoki et al., 2003). Studies with mouse *Dom* mutants, which possess a frameshift mutation in *Sox10* (Herbarth et al., 1998), demonstrate that *Sox10* is required for melanocyte development. Dom mutants lack expression of *Dct/Trp2*, an early melanocyte marker (Southard-Smith et al., 1998). Supporting these findings, *Sox10*^*LacZ/+*^ heterozygous mice have fewer melanocytes and *colorless* zebrafish mutants (premature stop codon in *sox10*) have reduced melanocytes, iridoblasts, and xanthoblasts (Kelsh and Eisen, 2000; Britsch et al., 2001; Dutton et al., 2001). Overexpression of *sox10* in *Xenopus* embryos results in a massive expansion of melanocyte precursor cells and is sufficient to induce the expression of melanocyte marker *dct/trp2* in naïve ectoderm (Aoki et al., 2003). Likewise, expression of *sox9* produces supernumerary melanocytes in *Xenopus* embryos (Taylor and LaBonne, 2005).

In the GRN controlling melanocyte development Sox10 has been shown to directly activate *Mitf*, *Dct/Trp2*, *Tyr*, and *Tyrp1* (Bondurand et al., 2000; Potterf et al., 2000; Jiao et al., 2004; Murisier et al., 2006, 2007). SOX10 acts synergistically with PAX3 to activate *MITF* expression (Bondurand et al., 2000; Potterf et al., 2000). Subsequently, MITF becomes a transcriptional partner for SOX10 and together they promote *DCT/TRP2* expression (Jiao et al., 2004; Ludwig et al., 2004). In the absence of *Sox10*, MITF alone is unable to direct formation of pigmented cells (Hou et al., 2006). Additionally, BRG1, a key member of the chromatin remodeling SWI/SNF complex, regulates proximal and distal promotor accessibility of melanocyte-specific SOX10 target genes. SOX10 directly interacts with BRG1, possibly recruiting BRG1 to these sites (Marathe et al., 2017). Global analysis of SOX10 binding sites in an immortalized melanocyte cell line further indicates that SOX10 binds to target sites as either a monomer or homodimer and most of these sites were found at distal regulatory regions. Interestingly, target genes are both up and downregulated in *Sox10*^*LacZ/+*^ cells providing further evidence that SOX10 can have both activator and repressor functions (Fufa et al., 2015). Validating and integrating these targets into the existing melanocyte GRN will greatly advance our understanding of melanocyte formation and disease etiology for Waardenburg Syndrome.

SOX10 activity during melanocyte formation is regulated in several ways. First, SOX5 can bind to Sox10 target regions within promotors of melanocyte genes and recruit the transcriptional corepressors HDAC1 and CtBP2 (Stolt et al., 2008). Although it has not been demonstrate that competition for binding sites occurs *in vivo*, *Sox5^–/–^, Sox10^+/lacZ^* double mutant embryos display a less severe melanocyte phenotype than *Sox10*^+/lacZ^ single mutant embryos, consistent with a role for SOX5 as a recruiter of corepressors (Stolt et al., 2008). Post-translational modification of SOX10 may also regulate its function during melanocyte formation. SOX10 can be SUMOylated at three lysine residues and this modification represses transcriptional activation of the *MITF* promotor *in vitro* (Taylor and LaBonne, 2005; Girard and Goossens, 2006).

In addition to regulating melanocyte formation during embryonic development, Sox factors are also integrally involved in postnatal melanocyte maintenance and progression of melanoma. A population of melanocyte stem cells resides at the base of hair follicle cells and contributes to the pigmentation of each hair shaft. *Sox10* is expressed in these stem cells and gain and loss of *Sox10* function both lead to a reduction in this cell population and the presence of white/gray hairs, consistent with disruptions in melanocyte stem cell function (Harris et al., 2013). Additionally, *Sox10* is expressed in the differentiated melanocytes of hair follicles and is required for retention of melanocytes (Harris et al., 2013). *SOX9* is expressed in postnatal melanocytes and can induce expression of SOX10 melanocyte target genes in B16 melanoma cells. Additionally, increased expression of *SOX9* leads to enhanced melanin production (Passeron et al., 2007). Together these findings suggest a SOX9 function in adult melanocytes that parallels that of SOX10 during embryonic development. Finally, both *SOX9* and *SOX10* have roles in the etiology of melanoma. While a detailed description of their functions in this context is beyond the scope of this review, we highlight that these SoxE factors may have antagonistic roles in melanoma cells (Shakhova et al., 2015). Sox functions in various cancers has recently been reviewed (Grimm et al., 2019).

#### Sox Factors and the Peripheral Nervous System

Neural crest cells also give rise to much of the peripheral nervous system (PNS), including the cephalic ganglia, dorsal root ganglia (DRG), Rohon-Beard cell, satellite cells, and Schwann cells (D’Amico-Martel and Noden, 1983; LeDouarin and Kalcheim, 1999). *Sox2, Sox4*, *Sox5*, *Sox10*, and *Sox11* are each expressed in various neural crest-derived PNS cells (Britsch et al., 2001; Perez-Alcala et al., 2004; Aquino et al., 2006). While functions for *Sox2*, *Sox4*, and *Sox10* have been described, only expression data for *Sox5* and *Sox11* has been reported (Figure 3). Schwann cell precursors, satellite glia, myelinating/non-myelinating cells, peripheral glia, and NC-derived cells within trigeminal ganglion express *Sox5* (Perez-Alcala et al., 2004; Morales et al., 2007). *Sox11* is expressed in several PNS cell types including DRG, cranial ganglia, and sympathetic ganglia (Hargrave et al., 1997; Kuhlbrodt et al., 1998a; Sock et al., 2004). Expression in these regions was noted to decrease over time and was very weak by e15.5 of mouse embryogenesis, suggesting that Sox11 may function in fate determination or early stages of differentiation (Hargrave et al., 1997; Kuhlbrodt et al., 1998a; Sock et al., 2004). Another SoxC subfamily member, Sox4, may possibly have an opposing role in PNS development and act as a negative regulator of Schwann cell myelination. Sox4 overexpression in Schwann cells leads to delayed myelination and hypomyelination. Interestingly, *Sox4* expression is elevated in mouse models of demyelinating neuropathies (Bartesaghi et al., 2015). These Sox11 and Sox4 findings provide evidence that members of the same Sox subfamily can play divergent roles.

Sox2, which is critical for maintaining stem-cell attributes in central nervous system progenitors, also functions in a subset of PNS cells (Wakamatsu et al., 2004; Le et al., 2005; Pevny and Placzek, 2005; Aquino et al., 2006; Adameyko et al., 2012). *Sox2* is expressed in neuroglial progenitors cells, but is downregulated upon differentiation (Aquino et al., 2006). *In ovo* electroporation of *SOX2* in chick embryos results in increased proliferation of DRG cells, but blocks neuroglial progenitor differentiation to both neural and glial fates (Wakamatsu et al., 2004) while knockdown/knockout of Sox2 in the DRG neural progenitors reduces the number of DRG neurons (Cimadamore et al., 2011). Together, these data indicate that SOX2 is an essential regulator of sensory neurogenesis. In addition, SOX2, is expressed in immature Schwann cells where it suppresses expression of genes associated with Schwann cell myelination and blocks myelination, an indicator of Schwann cell differentiation and maturation (Le et al., 2005; Roberts et al., 2017; Wust et al., 2020). It is thus clear that Sox2 plays critical roles in both the developing PNS and CNS.

Importantly, the SoxE factor, *Sox10*, is also a critical regulator of glial cell development. *Sox10* is expressed in Schwann cell precursors and sensory ganglia, and is required for the specification of all glia within the PNS (Kuhlbrodt et al., 1998b; Southard-Smith et al., 1998; Britsch et al., 2001). In the absence of *Sox10* (*Sox10*^*lacZ*^/*Sox10*^*lacZ*^ or *colorless*) cranial ganglia, enteric ganglia, and DRG numbers are reduced or display aberrant cell morphology (Kelsh and Eisen, 2000; Britsch et al., 2001; Dutton et al., 2001) and Schwann cell precursors are absent in both mutants (Kelsh and Eisen, 2000; Britsch et al., 2001; Dutton et al., 2001). These phenotypes are reminiscent of loss of neuregulin/ErbB signaling, which promotes the differentiation of neural crest into glia (Britsch et al., 2001; Britsch, 2007). *ErbB3* expression is decreased in *Sox10*^*lacZ*^/*Sox10*^*lacZ*^ mutants and, subsequently, migration and survival of progenitor cells is compromised (Britsch et al., 2001). In addition to regulating expression of *ErbB3*, *in vitro* studies have demonstrated that SOX10 synergies with OCT6 (POU3F1) and BRN2 (POU3F2) to activate *EGR2* (*KROX-20*), which is essential for myelination of Schwann cells (Kuhlbrodt et al., 1998b; Le et al., 2005). SOX10 then partners with EGR2 to activate others myelin genes (LeBlanc et al., 2007). Indeed, SOX10 and EGR2 are sufficient to reprogram skin fibroblasts into Schwann cells, emphasizing the importance of both of these factors for Schwann cell development (Mazzara et al., 2017). Regulation of Sox10 in Schwann cells has been linked with eEF1A1 which, upon acetylation, removes SOX10 from the nucleus. In Schwann cells, this activity is blocked by HDAC1/2, Sox10 co-factors essential for myelination, which deacetylate eEF1A1 causing it to return to the cytoplasm and preventing nuclear export of Sox10 (Duman et al., 2020). Finally, one of the essential functions of Sox10 is to direct the neuroglial progenitor cells of the DRG toward the glial lineage. Mechanistically this is attributed to Sox10 biasing neuroglia progenitor cells toward the glial lineage (vs. sensory neurons) by promoting the ubiquitination and subsequent degradation of transcription factor Neurog2 through upregulation of Fbxo9 (Liu et al., 2020).

### Craniofacial Structures

#### Palatogenesis

Cleft lip/palate is one of the most common birth defects, and numerous genes have been associated with this congenital malformation (Dixon et al., 2011). Mammalian palatogenesis begins with proliferation of the neural crest-derived cells within the palatal shelves, which leads to vertical outgrowth. The palatal shelves then elevate, sitting horizontal above the tongue, and the epithelium of the two shelves fuse. The epithelial seam formed at the midline is then removed to create a confluent mesenchyme (Bush and Jiang, 2012). A defect in any of these steps can result in a cleft secondary palate. Of the Sox transcription factors, only *SOX11* has been associated with a patient presenting with cleft palate (Khan et al., 2018). Nevertheless, several Sox mutant mice (*Sox11^–/–^, Sox9^*fl/fl*^;Wnt1-Cre*, *Sox2*^*HYP*^, *Sox5*^–/–^) develop cleft palates (Smits et al., 2001; Mori-Akiyama et al., 2003; Sock et al., 2004; Langer et al., 2014). During e12.5-e14.5 of murine palatogenesis, expression of *Sox2*, *Sox4*, *Sox5*, *Sox6*, *Sox8*, *Sox9*, *Sox11*, *Sox12*, *Sox13*, and *Sox21* can be detected via *in situ* hybridization (Table 1; Watanabe et al., 2016). *Sox2* is expressed in the epithelium; *Sox4/5/8/9* are expressed in the mesenchyme, while *Sox6/11/12/13* are expressed in both the palatal mesenchyme and epithelium (Watanabe et al., 2016). Of note, both *Sox2* and *Sox11* are expressed in the palatal epithelial seam at e14.5. While it is unclear if/how Sox factors regulate palatogenesis, the spatio/temporal expression patterns make it tempting to speculate that several factors play roles in this process.

#### Odontogenesis

Tooth development begins as the oral epithelium thickens and the underlying neural crest-derived mesenchyme condenses around the invaginating placode. The placode continues to elongate (bud stage) and then branch (bell stage). Subsequently, cells begin to differentiate into ameleoblasts and odontoblasts (bell stage) and terminally differentiate/mineralize just prior to root formation and eruption (Thesleff, 2014). Several Sox factors are expressed throughout odontogenesis (see Kawasaki et al., 2015 for detailed expression analysis); however, functional roles for most factors have not been described (Table 1). *Sox2* is expressed in the oral epithelium beginning at tooth initiation and continues to be expressed through cap stages in the lingual bud epithelium (Juuri et al., 2012; Kawasaki et al., 2015). Conditional deletion of *Sox2* from the oral epithelium (*Sox2^*fl/fl*^;Shh:GFP-Cre*) results in only minor defects in the second and third molars; however, recombination of *Shh:GFP-Cre* was mosaic and some SOX2 protein still detectable (Juuri et al., 2013). In contrast, conditional deletion of *Sox2* from both the oral and dental epithelium (*Sox2^*fl/fl*^;Pitx2-Cre*) produces abnormally shaped molars and underdeveloped incisors that regressed until undetectable at P0 (Sun et al., 2016). The regression of the incisors is consistent with a role for SOX2 in dental epithelial stem cells (DESCs). Lineage tracing experiments have revealed that Sox2 + DESCs reside in the labial cervical loop and contribute to all epithelial lineages in the mouse incisor (Juuri et al., 2012). Ablation of *Sox2* prior to incisor injury dramatically decreases the ability of the incisor to regrow (Sun et al., 2016). These mutants also display reduced proliferation in labial cervical loops, suggesting that SOX2 regulates DESC proliferation (Sun et al., 2016). In addition to murine DESCs, Sox2 defines an analogous stem cell population in cartilaginous fish, which regenerate teeth successionally, suggesting that SOX2 function in DESCs is evolutionarily conserved (Martin et al., 2016).

#### Salivary Gland Development

Three pairs of salivary glands, the sublingual (SL), the submandibular (SMG), and the parotid (PG), reside inside the oral cavity and together secrete up to a quart of saliva daily (Knosp et al., 2012). Embryonically, these structures begin as placodes within the oral epithelium and then subsequently undergo elongation and branching morphogenesis (Affolter et al., 2003). *Sox9*, *Sox10*, and *Sox2* are all expressed during salivary gland development (Lombaert et al., 2013; Chatzeli et al., 2017; Emmerson et al., 2017). *Sox9* is expressed in the oral epithelium that gives rise to the SMG, SL, and PG. Sox9^+^ epithelial cells serve as the progenitor cells for the entire epithelial component of these salivary glands (Chatzeli et al., 2017). As SMG/SL/PG development progresses *Sox9* becomes restricted to the distal progenitor cells of the bud. Conditional deletion of *Sox9* within the oral epithelium (*Sox9^*fl/fl*^;K14-Cre*) results in arrested SMG, SL, and PG development at the bud stage (Chatzeli et al., 2017). In the conditional mutants, these cells fail to become specified; however, the proximal progenitors (*Sox9*^–^) are specified normally (Chatzeli et al., 2017). In the absence of distal progenitor cells, branching morphogenesis fails to occur. Embryonic expression of *Sox9* ceases during lumen formation, but *Sox9* becomes expressed again in the adult and contributes to regulation of the stem/progenitor cell properties of a subpopulation of salivary gland cells (Chatzeli et al., 2017; Tanaka et al., 2019). *Sox10* is expressed in the distal progenitor cells of the bud and is also an acinar (mucin producing cells) progenitor marker (Lombaert et al., 2013). *Sox10* lies downstream of Sox9, Sox2, Kit, and FGF signaling in these cells (Lombaert et al., 2013; Chatzeli et al., 2017; Emmerson et al., 2017). While a specific function for Sox10 within the distal/acinar progenitor cells has not been determined, it is known to be essential for branching and acinar formation of other glands (Chen et al., 2014). *Sox2* is expressed in both duct and acinar progenitor cells but is only required for acinar cell formation. Sox2 promotes the expression of acinar-specific genes, including *Sox10*, and promotes survival of acinar progenitor cells through both maintaining proliferation and preventing apoptosis. Interestingly, parasympathetic nerves are required to maintain the Sox2 + progenitor cells, and thus are necessary for acinar cell formation (Emmerson et al., 2017).

## Soxopathies With Associated Craniofacial Phenotypes

Given the importance of Sox factors to the formation of the vertebrate craniofacial complex, it is perhaps unsurprising that a number of human syndromes presenting with craniofacial defects are linked to mutations in *SOX* genes (Table 2).

**TABLE 2:**
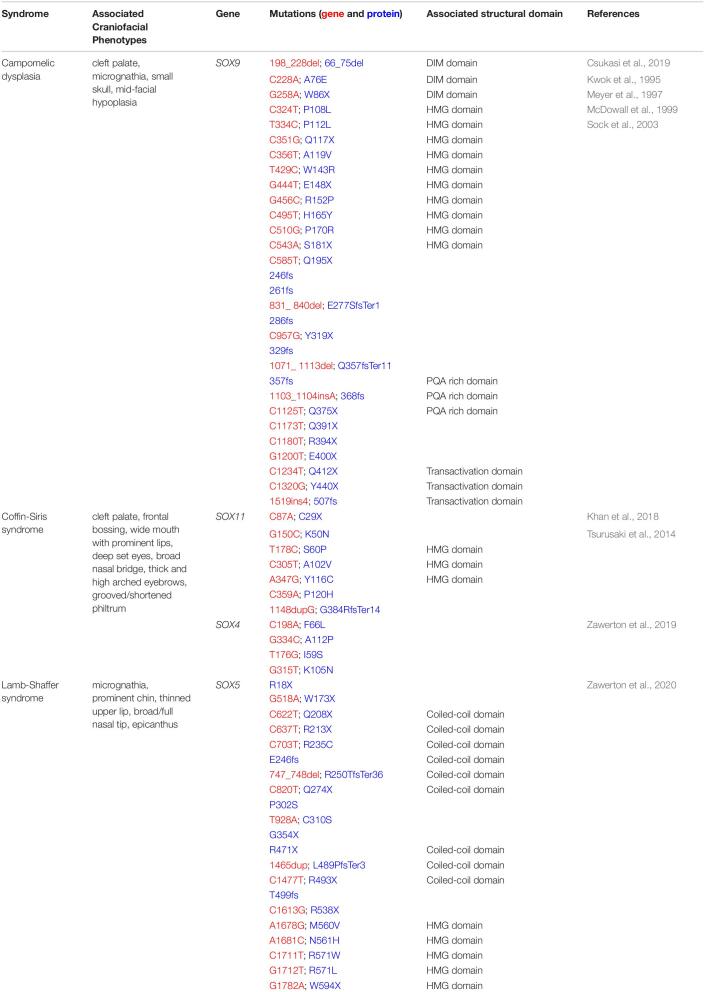
SOXopathies with craniofacial phenotypes.

### Campomelic Dysplasia

One of the most dramatic of these SOXopathies is Campomelic dysplasia (CD) which is caused by mutations in *SOX9* (Foster et al., 1994; Wagner et al., 1994). Individuals with CD typically die shortly after birth and display an undermineralized skeleton and dramatic bowing of the lower limbs. Within the craniofacial complex, common CD phenotypes include cleft palate, micrognathia, a small skull, and mid-facial hypoplasia (Csukasi et al., 2019). Most CD cases are caused by a *de novo* mutation within a *SOX9* allele (autosomal dominant). While the exact mutations within the *SOX9* gene varies among CD patients, there is evidence for both haploinsufficiency and dominant negative protein function underlying the observed phenotypes (Foster et al., 1994; Csukasi et al., 2019), and in mice, loss of one allele of *Sox9* phenocopies CD (Bi et al., 2001). Cell culture studies have shown that both *SOX9* haploinsufficiency and dominant negative forms of SOX9 (nonsense mutations in the C-terminal transactivation domain) fail to robustly activate *Col2a1* gene expression indicating that the chondrogenic program is not being fully initiated in cases of CD (Csukasi et al., 2019). Notably, although it is dysmorphic, a majority of the skeleton still forms in CD patients and animal models. Perhaps most SOX9 target genes are still activated despite loss of one functional allele. Alternately, SOX5 and SOX6 have partial functional redundant functions to SOX9 during chondrogenesis and may be able to compensate.

### Waardenburg Syndrome

Waardenburg syndrome (WS) is a neurocristopathy characterized by pigment abnormalities in the hair, skin, and eyes, hearing loss, and craniofacial alterations such as hypertelorism, dystopia cantorum, nasal hypoplasia, and harelip (Banerjee, 1986; Dourmishev et al., 1999; Pingault et al., 2010; Wildhardt et al., 2013; Chen et al., 2015). There are four different subtypes of WS, two of which are associated with mutations in *SOX10*, type 2 and type 4 (Pingault et al., 1998; Southard-Smith et al., 1999; Bondurand et al., 2007). Individuals with type 2 present with additional neurological defects while those with type 4 also have Hirschsprung’s disease (Bondurand et al., 2007). Over 40 different *SOX10* mutations have been reported across WS patients. Many mutations are truncating, causing the *SOX10* transcript to undergo nonsense-mediated RNA decay resulting in a phenotype driven by haploinsufficiency (Inoue et al., 2004). A few variants have also been reported that alter the *SOX10* stop codon and extend the protein (Pingault et al., 2010). For these variants, there is *in vitro* evidence that these elongated proteins have dominant negative effects (Inoue et al., 1999; Sham et al., 2001; Chan et al., 2003). During normal embryonic development, SOX10 is essential for neural crest stem cell formation and then subsequently for formation of specific derivatives, including melanocytes and enteric ganglia neurons (Southard-Smith et al., 1998; Britsch et al., 2001; Dutton et al., 2001). SOX10 also promotes survival and proliferation of Schwann cells (Britsch et al., 2001). In addition to *SOX10*, mutations in *PAX3*, *MITF*, *SNAI2*, *EDN3*, and *EDNRB* have all been identified as causative genetic insults for Waardenburg syndrome (Pingault et al., 2010). Together with SOX10, these genes are components of the gene regulatory networks controlling melanocyte (*PAX3*, *MITF*, *SNAI2*) or PNS (*EDN3, EDNRB*) development (Bondurand et al., 2007). Thus, WS patients with mutations in different genes can present with the same disease etiology due to disruptions in a shared gene regulatory network.

### Lamb-Shaffer Syndrome

Lamb-Shaffer syndrome (LAMSHF) is classified as a neurodevelopmental disorder with common phenotypes including: developmental delays, intellectual disability, and language/motor deficits (Lamb et al., 2012). Patients also have a signature set of craniofacial features: micrognathia, prominent chin, thinned upper lip, broad/full nasal tip, and epicanthus (Zawerton et al., 2020). Mutations in *SOX5*, a member of the SoxD family, have been linked with LAMSHF. The observed skeletal defects such as micrognathia, broad/full nasal tip, and prominent chin are consistent with SOX5 having a role in chondrogenesis. *Sox5* is strongly expressed in Meckel’s cartilage in mice (Ishikawa et al., 2018), which could explain the presentation of micrognathia, specifically. The general lack of severity of these craniofacial phenotypes is most likely due to individuals possessing other functional SOXD alleles, which is supported by *Sox5*^–/–^ having only minor skeletal defects (Smits et al., 2001). *In vitro* studies suggest that haploinsufficiency, rather than a dominant negative effect associated with SOX5 variants, are most likely causative of LAMSHF. Furthermore, SOX5 variants with nonsense mutations or missense mutations within the HMG were localized cytoplasmically, unable to bind DNA, and failed to activate gene expression. While molecular studies demonstrated that some SOX5 variants could still activate target gene expression and other variants could not, the study could not identify any genotype-phenotype correlation among LAMSHF patients (Zawerton et al., 2020).

### Coffin-Siris Syndrome

Another syndrome that has been associated with mutations in *SOX* genes, specifically Sox C family members is Coffin-Siris syndrome (CSS). Individuals with CSS have fifth fingers with clinodactyly, nail hypoplasia, microcephaly, and intellectual disabilities. Craniofacial features include cleft palate, frontal bossing, wide mouth with prominent lips, deep set eyes, broad nasal bridge, thick and high arched eyebrows, and a grooved/shortened philtrum (Tsurusaki et al., 2014; Hempel et al., 2016; Khan et al., 2018; Okamoto et al., 2018). Most patients with CSS (55–70%) have mutations in genes that encode for subunits of the BAF complex (Tsurusaki et al., 2014). Of the remaining cases, mutations in *SOX11* have been identified as causal for several unrelated patients (Tsurusaki et al., 2014; Hempel et al., 2016; Khan et al., 2018; Okamoto et al., 2018). Most of the identified mutations in *SOX11* lie within the HMG domain and result in decreased transcription of SOX11 target genes *in vitro* (Tsurusaki et al., 2014; Hempel et al., 2016). One variant has a mutation outside of this domain that is predicted to produce a truncated, non-functional protein (Khan et al., 2018). Additionally, there have been four cases of CSS where heterozygous mutations in *SOX4* have been identified. Like SOX11, these mutations were within the HMG domain and variant proteins were unable to bind DNA and activate target gene expression (Zawerton et al., 2019). Little is known about the roles of SoxC factors in the context of craniofacial development. *Sox4* and *Sox11* are broadly expressed in the neural crest-derived facial mesenchyme, and *Sox11* is, interestingly, expressed in the palatal epithelial seam (Watanabe et al., 2016). Whether Sox11 is functionally important for the removal of the epithelial seam is unknown, but this could explain occurrences of cleft palate in some CSS patients. In other cellular contexts, *Sox4* and *Sox11* promote proliferation (Gadi et al., 2013; Dai et al., 2017), thus it is possible that they may be regulating proliferation to some extent within the facial mesenchyme. Misregulation of proliferation could lead to phenotypes such as broad nasal bridge, shortened philtrum, and prominent lips. In the future, it would be interesting to use animal models to study the effects of single or combined loss of *Sox4* and *Sox11* on craniofacial development.

### Hypotrichosis-Lymphedema-Telangiectasia Syndrome

Lastly, mutations in *Sox18* have been identified in patients with a rare condition called Hypotrichosis-lymphedema-telangiectasia syndrome (HLTS) (Irrthum et al., 2003). As the name suggests, the predominating features of these patients are sparse hair, tissue swelling due to malfunctioning lymphatic system, and presence of dilated vessels skin surface. While most case studies do not report a craniofacial phenotype with HLTS (either no phenotype or not assessed), a few patients have mild craniofacial defects that include: thick lips, microcephaly, periorbital swelling, and broad nasal tip (Bastaki et al., 2016; Valenzuela et al., 2018; Wangberg et al., 2018). Unlike the other syndromes associated with mutations in *SOX* genes, HLTS is associated with both autosomal dominant and autosomal recessive modes of inheritance (Irrthum et al., 2003; Wangberg et al., 2018). The autosomal dominant mode of inheritance is typically associated with nonsense mutations in the SOX18 transactivation domain while the autosomal recessive form is marked by missense mutations within the HMG domain (Valenzuela et al., 2018). Expression data in mice indicates that *Sox18* primarily localizes to sites of vascularization within the developing murine craniofacial complex (Ishikawa et al., 2018). Given this expression, it is unclear how loss of *Sox18* could result in phenotypes such as microcephaly or broad nasal tip. Clearly, further study into the molecular mechanisms underlying this syndrome, specifically those associated with the craniofacial defects, is necessary.

## Conclusion and Perspectives

The emergence of neural crest drove the evolution of vertebrates including the elaboration of an intricate craniofacial complex. While Sox transcription factors are heavily utilized during invertebrate development, new roles have evolved for many of these factors within vertebrate cell types and structures, aided by duplication events. SoxE factors are initially required for the formation of neural crest stem cells, analogous to the role that SoxB1 factors play in blastula (inner cell mass) stem cells. Subsequently, SoxE factors are essential for the diversification of neural crest cells into a subset of non-neural lineages including cartilage, melanocytes and glia. By contrast SoxB1 factors transition to maintaining a neural progenitor state, and some SoxB1 partner pairings, including POU factors, are maintained between blastula stem cells and neural progenitor cells. Conserved SoxB1 roles within these cell populations could have necessitated the deployment of a different Sox subfamily, SoxE, in neural crest progenitors and derivatives. Evolutionarily, SoxE factor duplications at the base of the vertebrates may have helped drive neural crest lineage diversification and the development of the vertebrate craniofacial complex. Understanding the role of Sox proteins in the emergence of specialized cell types and complex forms in vertebrates will require a fuller understanding of the shared and unique functions of different Sox factors and families, and the mechanisms regulating those functions. This includes defining their transcriptional targets in different cellular contexts. Such studies should also prove to be of high clinical significance given the many congenital defects associated with Sox mutations. In particular, the plethora of craniofacial phenotypes associated with SOXopathies underscores the critical roles these factors play in the development and evolution of the vertebrate craniofacial complex.

It has been 30 years since the discovery of the SRY gene yet we are continuously learning more about the roles and regulation of this important family of transcription factors. Within the context of the neural crest and craniofacial complex, there are several outstanding questions that are ripe for study. Sox9 has been shown to have pioneer activity in hair follicle stem cells (Adam et al., 2015); however, it is unknown whether this function extends to the neural crest. One intriguing possibility is that Sox9 and other SoxE factors, through pioneer activity, set the stage in the chromatin landscape of neural crest progenitors for the subsequent adoption of specific lineage states. Recent data has shown that the chromatin of vagal neural crest is biased toward specific lineages prior to the onset of migration (Ling and Sauka-Spengler, 2019). It will be important to determine if such biases also exist in cranial neural crest and if different SoxE factors play roles in establishing these predispositions. In addition, there is evidence for direct interaction of Sox factors with epigenetic factors, such as HDACs, to regulate cell fate decisions (Duman et al., 2020). To date, studies of Sox partners/co-factors have predominately focused on other transcription factors. It is essential, however, to broaden our understanding of Sox interacting factors to include epigenetic modifiers, and to determine how these interactions shape the chromatin landscape within the neural crest and its derivatives. Finally, global histone acetylation in the neural crest differs from that of differentiated cells and actually more closely resembles that of blastula stem cells (Rao and LaBonne, 2018). To what degree are these similar epigenetic signatures mediated by Sox factors? Does switching regulation from SoxB1 in blastula stem cells to SoxE factors in neural crest stem cells lead to maintained chromatin architecture at Sox targets within the pluripotency GRN? As more large-scale sequencing experiments are conducted, and with the growing power of single cell approaches, such questions are likely to be answered in the near future.

## Author Contributions

ES wrote the manuscript. CL edited the manuscript. Both authors contributed to the article and approved the submitted version.

## Conflict of Interest

The authors declare that the research was conducted in the absence of any commercial or financial relationships that could be construed as a potential conflict of interest.
